# Introducing a multi-site program for early diagnosis of HIV infection among HIV-exposed infants in Tanzania

**DOI:** 10.1186/1471-2431-10-44

**Published:** 2010-06-17

**Authors:** Harriet Nuwagaba-Biribonwoha, Bazghina Werq-Semo, Aziz Abdallah, Amy Cunningham, John G Gamaliel, Sevestine Mtunga, Victoria Nankabirwa, Isaya Malisa, Luis F Gonzalez, Charles Massambu, Denis Nash, Jessica Justman, Elaine J Abrams

**Affiliations:** 1 Columbia University, International Centre for AIDS Care and Treatment Programs (ICAP), Dar es salaam, Tanzania; 2 Columbia University, International Centre for AIDS Care and Treatment Programs (ICAP), New York, USA; 3 Bugando Medical Centre Laboratory, Mwanza, Tanzania; 4 Ministry of Health and Social Welfare, Dar es Salaam, Tanzania; 5 International Training and Education Centre for Health in Botswana; 6 University Research Co., LLC-Centre for Human Services (URC-CHS) in Namibia; 7 Mylan Pharmaceuticals Inc., Tanzania

## Abstract

**Background:**

In Tanzania, less than a third of HIV infected children estimated to be in need of antiretroviral therapy (ART) are receiving it. In this setting where other infections and malnutrition mimic signs and symptoms of AIDS, early diagnosis of HIV among HIV-exposed infants without specialized virologic testing can be a complex process. We aimed to introduce an Early Infant Diagnosis (EID) pilot program using HIV DNA Polymerase Chain Reaction (PCR) testing with the intent of making EID nationally available based on lessons learned in the first 6 months of implementation.

**Methods:**

In September 2006, a molecular biology laboratory at Bugando Medical Center was established in order to perform HIV DNA PCR testing using Dried Blood Spots (DBS). Ninety- six health workers from 4 health facilities were trained in the identification and care of HIV-exposed infants, HIV testing algorithms and collection of DBS samples. Paper-based tracking systems for monitoring the program that fed into a simple electronic database were introduced at the sites and in the laboratory. Time from birth to first HIV DNA PCR testing and to receipt of test results were assessed using Kaplan-Meier curves.

**Results:**

From October 2006 to March 2007, 510 HIV-exposed infants were identified from the 4 health facilities. Of these, 441(87%) infants had an HIV DNA PCR test at a median age of 4 months (IQR 1 to 8 months) and 75(17%) were PCR positive. Parents/guardians for a total of 242(55%) HIV-exposed infants returned to receive PCR test results, including 51/75 (68%) of those PCR positive, 187/361 (52%) of the PCR negative, and 4/5 (80%) of those with indeterminate PCR results. The median time between blood draw for PCR testing and receipt of test results by the parent or guardian was 5 weeks (range <1 week to 14 weeks) among children who tested PCR positive and 10 weeks (range <1 week to 21 weeks) for those that tested PCR negative.

**Conclusions:**

The EID pilot program successfully introduced systems for identification of HIV-exposed infants. There was a high response as hundreds of HIV-exposed infants were registered and tested in a 6 month period. Challenges included the large proportion of parents not returning for PCR test results. Experience from the pilot phase has informed the national roll-out of the EID program currently underway in Tanzania.

## Background

There is increasing availability of antiretroviral treatment (ART) for adults and children in Tanzania but by December 2008, only 13,400 (32%) of the 42,000 children estimated to be eligible for ART were receiving treatment [1,2]. Infants and young children with HIV infection are at high risk for rapid disease progression and death [3-6], implying an urgent need to identify and enroll them into HIV care and treatment programs so that ART can be started at the earliest opportunity. Unfortunately, simple antibody-based diagnosis of HIV infection in infants is complicated by passive transfer of maternal HIV antibodies during pregnancy. More sophisticated molecular biologic tests such as those using polymerase chain reaction (PCR) technology are needed to distinguish HIV infected from HIV exposed but uninfected children during the first 1-2 years of life [7-9]. Clinical and immunologic criteria can be used for presumptive diagnosis of HIV infection for the purpose of starting ART [10], but these have low sensitivity and specificity [11,12] and symptoms of HIV infection can be difficult to distinguish from those of other prevalent conditions in uninfected children such as malnutrition and tuberculosis.

**Figure 1:**
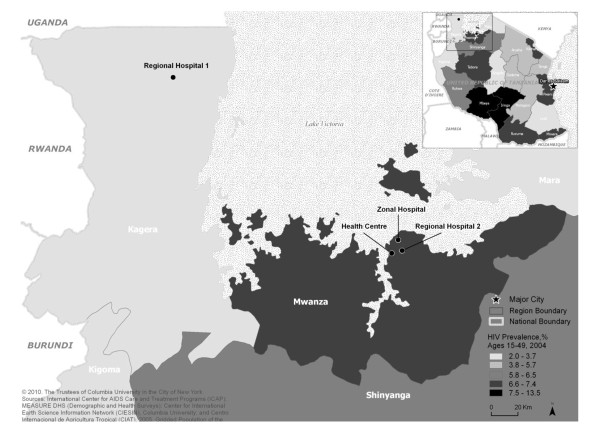
**Map of Tanzania showing the location of the Early Infant Diagnosis (EID) pilot program activities**.

Until recently laboratory capacity for program-level PCR-based assays has been limited in Sub-Saharan Africa and lacking in Tanzania, resulting in a significant barrier to early infant diagnosis (EID) and timely initiation of ART in HIV-infected infants [10]. The increasing availability of these assays at lower prices and the high sensitivity of HIV DNA PCR testing using Dried Blood Spots (DBS) have made it feasible to develop national EID programs in several Sub-Saharan African countries [7,13-16]. We introduced a pilot EID program in the Lake region of Tanzania (Figure 1) aiming to establish laboratory capacity and infrastructure to perform HIV DNA PCR testing; and to develop clinical infrastructure and systems to support identification, care and follow-up of HIV-exposed infants in both remote rural and moderately urban settings. We report on the experience of developing and establishing the EID program, describe the characteristics of children served, and report on successes and challenges of the pilot phase in order to inform the on-going scale up of EID services in Tanzania and Sub-Saharan Africa.

## Methods

The EID program was collaboratively designed and implemented by the International Center for AIDS Care and Treatment Programs (ICAP), the Tanzanian Ministry of Health and Social Welfare, Bugando Medical Centre (BMC) and the United States Centers for Disease Control and Prevention (CDC). The steps taken in establishment of the EID program are described below and summarized in Table 1.

**Table 1:** Approaches to the critical processes of setting up the Early Infant Diagnosis (EID) program in Tanzania, outcomes observed and challenges encountered

Process	Approach	Outcomes observed	Challenges encountered
Community preparation:	-Engaged communities before services were introduced. -Targeted influential community leaders for community advocacy. -Utilized community gatherings for mass communication.	-Created anticipation for the services. -Community members encouraged to access services. -Quickly informed many community members.	-Community members expected same-day test results and not to have to return for results at a later visit.

Health facility selection:	-Selected sites with existing PMTCT* programs and maternal child health clinics.	-There was a ready need for EID** services which then easily integrated.	-Some PMTCT programs were not functioning optimally in providing HIV testing and counseling and PMTCT antiretroviral regimens. -Infant follow-up was not a consistent component of all PMTCT programs.

Health facility preparation:	-Sensitized all health facility staff to refer possible HIV-exposed infants for EID services. -Identified space where EID services would be offered. -Worked with health facility staff to create and implement a detailed practical plan on how services would be offered. -Implemented a system for transporting samples from the site to the laboratory and results delivery back to the site.	-Children were referred from many service delivery points. -Streamlined registration and follow-up of HIV-exposed infants. -Streamlined delivery of services. -Established sample transportation and results delivery systems.	-Health workers had multiple competing responsibilities. -Maintaining efficiency of the sample transportation and results delivery system.

Capacity building:	-Conducted didactic training complemented with on-site mentorship.	-Empowered health workers and allowed them to develop confidence to implement services. -Ensured supervision in the early phases of implementation. -Linked didactic training to implementation.	-Transfer of trained personnel to other departments, facilities or regions. Request for financial incentives by health workers.

Laboratory establishment:	-Renovated an existing zonal laboratory that served multiple sites within the catchment area. -Trained laboratory staff at a laboratory with established PCR facilities. -Provided expert mentorship and on-going regular supervision at the laboratory. -Conducted quality assurance assessments according to standard procedures.	-Minimized start-up costs. -Laboratory staff gained a practical view of how systems work. -Ensured continuity of quality PCR services.	-Ensuring continuous supply of materials and supplies for DNA PCR testing. -Lab personnel had competing responsibilities.

Defining the HIV testing algorithm:	-Done at national level with involvement of key stakeholders including the Ministry of Health and donors.	-Promoted national and stakeholder acceptance of the testing algorithm.	-Reaching agreement on an algorithm that was both cost-effective but clinically relevant.

Registration and follow-up HIV-exposed infants and data collection:	-Created specific tools for recording data related to HIV-exposed infants and PCR testing. -Developed standard operating procedures for the new clinical services. -Registered HIV-exposed infants prior to availability of EID services were available. -Provided comprehensive follow-up care (e.g. giving cotrimoxazole). -Due to limited resources, preferentially followed-up infants that tested DNA PCR positive through appointment cards, phone calls and home visits.	-Monitoring and evaluation systems were readily available for the national roll-out. -Ensured HIV-exposed infants were being identified, registered and provided with necessary services. -Ensured that those at the greatest risk of mortality (HIV-infected infants) were referred to the HIV care and treatment clinic.	-Health services poorly equipped to retain infants for longitudinal follow-up. -High rate of loss to follow-up to obtain for DNA PCR results. -Some mothers stopped breastfeeding after first negative PCR result. -High rate of loss to follow-up to obtain final infection status after breast feeding cessation.

*PMTCT = Prevention of Mother to Child HIV Transmission; **EID = Early Infant Diagnosis

### Community preparation and site selection

Prior to program initiation, community and religious leaders as well as health officials at the district (e.g. District Medical Officers, District Reproductive Health Services Coordinator and District AIDS Coordinators) and regional levels (Regional AIDS Coordinator) were sensitized through meetings at which the planned EID program was explained by ICAP staff. These staff also spoke at church and mosque gatherings to inform the communities about the availability, importance and benefits of the EID program.

Four health facilities were purposively selected to reflect a variety of settings and included two regional hospitals, one zonal hospital which also housed the PCR laboratory, and one health centre in the Lake region of Tanzania (Figure 1). The first regional hospital was 208 km away from the laboratory, the second regional hospital was 8.6 km away and the health centre 11 km away. The selected health facilities had antenatal care clinics with well-established Prevention of Mother-to-Child HIV Transmission (PMTCT) programs where in total more than 7,000 pregnant women were counseled and tested for HIV annually following the national and World Health Organization (WHO) PMTCT guidelines at that time [17,18]. The 2 regional hospitals and the zonal hospital had HIV care and treatment clinics (CTCs) within the hospital complex. HIV infected women from the health centre were referred to the nearby regional hospital (within 2 km) for CTC services.

### Health facility preparation and staff capacity building

Existing health facility staff working in PMTCT clinics, maternity wards, pediatric clinics and pediatric wards as well as CTCs were informed of the upcoming EID program and their input sought during the planning phase. Staff began to track and register HIV-exposed infants from the sites and the community several months prior to the availability of DNA PCR testing. At each of the 4 pilot sites, an EID room was identified where counseling and DBS sampling could be conducted. At the regional hospitals and the health centre, the EID room was co-located within the antenatal PMTCT clinic, while at the zonal hospital; it was situated within the pediatric ward. Health facility staff were instructed to refer all HIV-exposed infants from their service delivery points to the EID room for DBS sampling. Each health facility defined patient flow patterns as a strategy to maximize identification, registration and testing of HIV-exposed infants.

A total of 96 health workers from the 4 health facilities including nurses, counselors, community health workers, clinicians, and laboratory staff received 2-day didactic training followed by 2 weeks of on-site supervision and mentorship. The training curriculum included identification of HIV-exposed infants, the HIV testing algorithm and interpretation of PCR results, DBS collection and the use of a paper-based data collection and monitoring system. Elements of HIV-exposed infant care including cotrimoxazole prophylaxis, growth monitoring, infant feeding, long-term follow-up until determination of final infection status and referral to CTCs for those HIV infected were emphasized during the training.

### Laboratory establishment

A molecular biology laboratory at BMC was established by renovation of the existing clinical laboratory and installation of PCR equipment. Two BMC lab staff received 4 weeks of training at the Government of Rwanda's National Reference Laboratory in Kigali followed by 4 weeks on-site supervision by an experienced laboratory advisor. Roche Amplicor v 1.5 DNA PCR kits (Roche Diagnostics, Inc., Alameda, CA) were procured and used as described by Sherman et al [7]. Internal quality control panels containing negative and high positive (5,000 proviral copies of HIV/ml) DBS were obtained from CDC's Global AIDS Program International Laboratory Branch in Atlanta. The results obtained were interpreted following the kit instructions. Every 3 months, all positive samples and 10% of the negative samples were re-tested for quality assurance. All samples with indeterminate results were repeat tested using the same sample, and an additional sample collected from the HIV-exposed infant at a follow-up visit. Data on HIV DNA PCR laboratory activities were recorded in a paper-based register and entered into a simple electronic (Microsoft Access) database.

### HIV testing algorithm and definitions

The HIV-exposed infant testing algorithm was determined in collaboration with the Government of Tanzania's Ministry of Health. Rapid HIV antibody screening was conducted on the infants of mothers with unknown HIV status. All children <18 months of age who either were HIV antibody positive or were born to a known HIV positive woman were defined as HIV-exposed infants. All parents and guardians of HIV-exposed infants were counseled on HIV DNA PCR testing and all HIV-exposed infants were tested unless the parent or guardian declined the test. The first DNA PCR testing was scheduled for 6 weeks of age, the time at which most infants in Tanzania received their first set of routine vaccinations. All HIV-exposed infants less than 18 months of age who had not had a previous HIV DNA PCR test were eligible for PCR testing. DBS were collected by heel, toe or finger stick, dried on a rack and then stored in an envelope with anti-humidity pellets according to standard procedures [7]. Infants testing positive at the first PCR test were defined as HIV-infected [19], had a DBS collected for a second (confirmatory) PCR test, and were referred to the CTC on the same day that the first PCR test results were given to parents or guardians. Infants testing PCR negative were followed clinically and tested again using HIV rapid antibody tests at least 6 weeks after weaning. Infants with initial negative PCR tests who developed clinical symptoms were eligible for repeat PCR testing.

### Registration, care and follow-up of HIV-exposed infants

HIV-exposed infants were registered in the EID room by recording key data in paper-based registers including a unique identifier, date of birth, gender, contact details, PMTCT prophylaxis taken by the mother and baby, HIV testing details and medications given. HIV-exposed infants who were 6 weeks or older were started on cotrimoxazole prophylaxis. Once a week, an EID nurse or courier service transported the DBS samples to the PCR laboratory and collected and delivered the previous week's results. Parents and guardians were given appointment cards and asked to return to the clinic 1 month after DBS collection to receive DNA PCR results and to refill cotrimoxazole prescriptions. If parents/guardians did not return to the clinic within 28 days of the scheduled appointment, contact tracing by phone and home visits was done by ICAP staff and health facility EID nurses in collaboration with community outreach groups. Due to the limited resources, staff prioritized visits to children testing PCR positive.

### Data collection and analysis

A paper-based system using longitudinal HIV-exposed infant registers and lab registers was implemented, and captured key information including date of birth, PMTCT prophylaxis, and HIV testing details. Standard Operating Procedures on how, when, and where to record the required data elements were availed to sites for reference. Data from the HIV-exposed infant registers were entered into an electronic database and updated monthly by ICAP staff. These routinely collected service delivery data from the HIV-exposed infant register database with updates through June 2007 were de-identified and analyzed using SAS version 9.1. The outcomes of interest were: time from birth to blood draw for the first HIV DNA PCR test, time from this blood draw to receipt of PCR test results by the parent/guardian and the proportion of parents/guardians receiving PCR test results. Characteristics of HIV-exposed infants were described in frequency tables and categorical variables were compared using chi-square statistics and time to events examined using stratified Kaplan-Meier curves. Parents/guardians that did not return for PCR test results were censored at the end of the data collection period in June 2007. This program was part of a larger HIV care and treatment program in Tanzania which received non-research determination from the Columbia University Institutional Review Board. Routinely collected data from the program were de-identified and analyzed for purposes of program evaluation so no consent was sought from participants.

## Results

From October 2006 to March 2007, 510 HIV-exposed infants (median age: 4 months, IQR: 1 to 8 months) were identified and registered. Table 2 summarizes the characteristics of these infants, 441 (86%) of whom were tested with DNA PCR. There were statistically significant differences between children who were tested with DNA PCR (n = 441) and the untested children (n = 69) on several characteristics. As expected because the testing algorithm recommended DNA PCR testing from 6 weeks, 90% of the untested children were less than 6 weeks of age at presentation, compared with 13% of those tested (p < 0.01). Ninety three percent of those untested were referred from PMTCT clinics and enrolled at one Regional Hospital. Tested children were more likely than those untested to initiate cotrimoxazole (98% vs 78%, p < 0.01), but more untested children and/or their mothers had received PMTCT prophylaxis, 91% vs 65% of those tested, p < 0.01. At the time of database closure, infant feeding data were incomplete and therefore excluded from this analysis.

**Table 2:** Characteristics of HIV-exposed infants tested and not tested for HIV by DNA PCR, and of infants testing PCR positive and PCR negative in the Tanzania Early Infant Diagnosis (EID) pilot program

	HIV-exposed infants not tested	HIV-exposed infants tested*	Total	**p-value**^§^	Infants with a positive first DNA PCR *	Infants with a negative first DNA PCR *	**p-value**^§^
	n = 69 (%)	n = 441(%)	n = 510 (%)		n = 75 (%)	n = 361 (%)	
**Gender**							
Male	16(23%)	252(57%)	268 (53%)	<0.01	45(60%)	206(57%)	0.82
Female	8(12%)	188(43%)	196 (38%)		30(40%)	154(43%)	
Missing data	45(65%)	1(0%)	46(9%)		0(0%)	1(0%)	
							
**Age at registration **^β^							
Less than 6 weeks	62(90%)	57(13%)	119 (23%)	<0.01	10(13%)	46(13%)	0.22
6 weeks - < 6 months	2(3%)	218(49%)	220 (43%)		36(48%)	179(50%)	
6 months - < 12 months	1(1%)	108(25%)	109 (21%)		14(19%)	93(26%)	
≥ 12 months	4(6%)	58(13%)	62 (12%)		15(20%)	43(12%)	
							
**Initiated cotrimoxazole prophylaxis**							
Yes	54(78%)	432(98%)	486 (95%)	<0.01	75(100%)	352(98%)	0.18
No	9(13%)	9(2%)	18 (4%)		0(0%)	9(2%)	
Missing data	6(9%)	0(0%)	6(1%)		0(0%)	0(0%)	
							
**Health facility where EID**^€ ^**services were received**							
Regional Hospital 1	0(0%)	93(21%)	93 (18%)	<0.01	14(19%)	76(21%)	0.75
Regional Hospital 2	59(86%)	122(28%)	181 (35%)		18(24%)	103(29%)	
Zonal Hospital	1(1%)	140(32%)	141 (28%)		27(36%)	112(31%)	
Health Centre	9(13%)	86(20%)	95(19%)		16(21%)	70(19%)	
							
**Referral source of HIV-exposed infants**							
HIV Care and Treatment Clinic (CTC)	2(3%)	205(47%)	207 (41%)	<0.01	35(47%)	169(47%)	0.11
PMTCT/Maternal Child Health clinics	64(93%)	197(45%)	261 (51%)		29(39%)	165(46%)	
Other	3(4%)	39(9%)	42 (8%)		11(15%)	27(7%)	
							
**PMTCT ARV's **^π ^**received by infants**							
Single-dose Nevirapine	60(87%)	270(61%)	330 (65%)	<0.01	34(45%)	232(64%)	<0.01
None	9(13%)	171(39%)	180 (35%)		41(55%)	129(36%)	
							
**PMTCT ARV's **^π ^**received by mothers**							
Highly Active Antiretroviral Therapy	1(1%)	11(2%)	12 (2%)	0.16	0(0%)	10(3%)	0.04
Single-dose Nevirapine	24(35%)	203(46%)	227 (45%)		27(37%)	173(48%)	
None	44(64%)	227(52%)	271 (53%)		48(64%)	178(49%)	
							
**Both mother and infant received PMTCT ARV's **^π^							
Yes	22 (32%)	198 (45%)	220 (43%)	0.04	25(33%)	169(47%)	0.03
No	47 (68%)	243 (55%)	290 (57%)		50(67%)	192(53%)	
							
**Mother and/or infant received PMTCT ARV's **^π^							
Yes	63(91%)	286(65%)	349(68%)	<0.01	36(48%)	246(68%)	<0.01
No	6(9%)	155(35%)	161(32%)		39(52%)	115(32%)	
							
**Parent/guardian received DNA PCR results**							
Yes	n/a	238(55%)*	n/a		51(68%)	187 (52%)	0.01
No		198(45%)			24(32%)	174(48%)	

*5 HIV-exposed infants with indeterminate results at first DNA PCR testing are excluded from this table, 4 of whom returned for PCR test results

^§ ^P-values are from Pearson's chi-square tests

^β ^Age that the status of being HIV-exposed was first recognised and formally recorded for follow-up

^€ ^EID = Early Infant Diagnosis

^π ^PMTCT ARV's = Prevention of Mother to Child HIV Transmission Anti Retroviral Therapy

**Figure 2:**
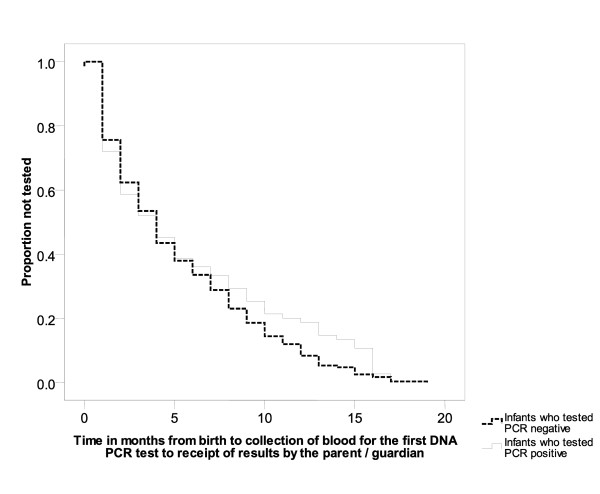
**Time in months from birth to first HIV DNA PCR test among HIV- exposed infants testing PCR positive and PCR negative in the Tanzania Early Infant Diagnosis pilot program**. The median age at first HIV DNA PCR testing was 4 months, IQR: 1 to 8 months, with no statistically significant differences in time to testing between HIV-exposed infants who tested PCR positive and those who tested PCR negative.

Among those tested (n = 441), the median age at first HIV DNA PCR testing was 4 months, IQR: 1 to 8 months, with no statistically significant differences in time to PCR testing between HIV-exposed infants who tested PCR positive and those who tested PCR negative (Figure 2). Of the 441 HIV-exposed infants tested, 75 (17%) were PCR positive, 361 tested PCR negative, and 5 (1%) had indeterminate test results (Table 2). The 5 infants with indeterminate first DNA PCR test results were tested at a median age of 7 weeks. Only 48% of the infants testing PCR positive were exposed to PMTCT prophylaxis (mother and/or infant had received ART for PMTCT) compared to 68% of those testing negative (p < 0.01).

**Figure 3:**
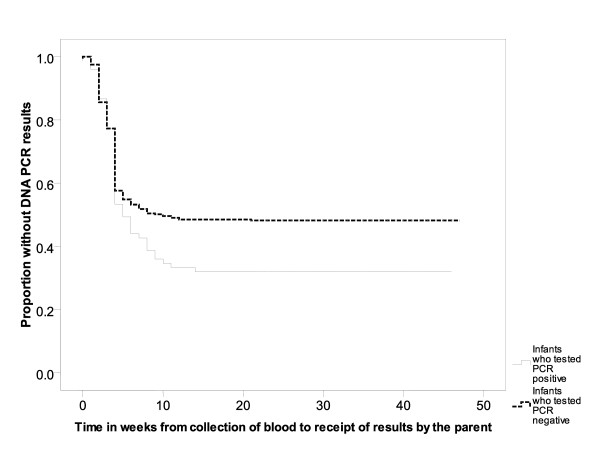
**Time in weeks from blood draw for HIV DNA PCR to parent receipt of results among HIV exposed infants testing PCR positive and PCR negative in the Tanzania Early Infant Diagnosis pilot program**. The median time between blood draw for PCR testing and receipt of test results by the parent or guardian was 5 weeks (range <1 week to 14 weeks) among HIV-exposed infants who tested PCR positive and 10 weeks (range <1 week to 21 weeks) for those that tested PCR negative.

A total of 242/441 (55%) HIV-exposed infants' parents/guardians returned for follow-up to learn the PCR test results, including 51/75 (68%) of the PCR positive children, 187/361 (52%) of the PCR negative children and 4/5 (80%) of those with indeterminate PCR results. The median time between blood draw for PCR testing and receipt of test results by the parent or guardian was 5 weeks (range <1 week to 14 weeks) among children who tested PCR positive and 10 weeks (range <1 week to 21 weeks) for those that tested PCR negative (Figure 3). Seven of the 52 DNA PCR positive infants whose parents/guardians received results had died by the time test results returned. Of the 45 infants who received results, 42 (93%) were referred to CTC, and all received a second DNA PCR test which was positive, confirming HIV infection. The other 3/45 infants died before they were referred to the CTC.

Of the 361 infants who tested DNA PCR negative, 108 (30%) were no longer breastfeeding at the time of testing. The other 253 children were expected to return for antibody testing after weaning, but only 42 (17%) had returned for testing by the time of this analysis. All but 3 (6.7%) of these 42 children tested antibody negative. The 3 who tested antibody positive also had a positive second DNA PCR test, which confirmed HIV infection. All the 4 children with indeterminate results at first DNA PCR testing who returned for results had a second DNA PCR test: 3 were PCR negative and 1 PCR positive at the second test.

## Discussion and Conclusion

This was the first service delivery program (outside of research initiatives) established for reliable HIV diagnosis among infants in Tanzania, and the only HIV DNA PCR service in the Lake Region. In addition to providing HIV diagnostic capacity, the introduction of PCR laboratory services stimulated the development and institutionalization of clinical programs for HIV-exposed infant follow-up. In the first 6 months of implementation, systems were established to identify HIV-exposed infants and engage them in care. This was a major success in a setting with no previous clear linkages between PMTCT programs and HIV-exposed infant follow-up, and no systems to diagnose HIV infection in infants prior to their presentation with symptoms of advanced HIV disease. The hundreds of HIV-exposed infants identified in a short period, 33% of whom were 6 months or older and the median age of testing at 4 months are a reflection of the back-log of un-tested infants in this community that lacked EID services prior to this program. Kenya observed a similar median age at PCR testing as services were rolled out [20] and similar high uptake of the program was observed in Botswana when the EID program was implemented in two large cities [13].

The first 6 months of implementing the EID program demonstrated other important successes. Life saving cotrimoxazole was initiated for the majority of the HIV-exposed infants enrolled in our EID program. There was a rapid turnaround time with many parents/guardian receiving PCR test results within a median of 5 weeks (PCR positive) and 10 weeks (PCR negative) after testing, demonstrating some similarities with what was observed in the Kenyan program [20]. This was facilitated by having a clearly outlined sample and results tracking and delivery system and was a significant improvement from the many months of waiting needed for the antibody test to reliably reflect HIV status. As programs scale up, less attention tends to be paid to this aspect leading to delay in return and utilization of results [21] and sample turn-around time can be a significant program barrier with long delays of several months. Another success was the development of EID standard operating procedures and training materials that gave guidance to the national EID program roll-out [22].

Challengingly, only 55% of the parents/guardians returned for PCR test results. Such significant attrition of HIV-exposed infants has been noted by other programs in sub-Saharan Africa [8,14,21,23], yet retaining children in care is essential to prevent mortality [24]. More parents and guardians of infants testing PCR positive returned for results likely because of the preferential follow-up by staff through community tracking and home visits. Also, more than 70% of children appeared to be still breastfeeding by the time of these analyses and the continued exposure to HIV warrants that children receive regular monitoring visits and repeat testing to determine final infection status. Program success depends on good systems to identify, follow, and retain all children in care. Additional research is needed to understand the reasons that prevent parents and guardians from returning for results and to determine optimal ways to continue to engage these families in HIV services.

The finding that overall 17% of HIV-exposed children tested DNA PCR positive on their first test is consistent with what has been observed in other programs where single dose Nevirapine was the primary PMTCT intervention [14,20,25]. However, it should be noted that 35% of infants and 53% of mothers were recorded as not having received any ARV intervention. Among the 282 tested babies who were exposed to either maternal and/or infant PMTCT ARVs 12.8% tested PCR positive, compared to 25.3% of the 154 infants where neither the mother nor the baby received PMTCT ARVs. Where PMTCT programs are better resourced and more efficacious PMTCT regimens are given, the proportion of HIV-exposed infants testing PCR positive is less than half what was observed in our program [13]. The challenge in Tanzania remains to increase uptake of ARV's for PMTCT and implement PMTCT programs utilizing multi-drug ART regimens which are more effective in reducing HIV transmission to infants. In addition, 6.7% of the 45 previously PCR negative children who returned for confirmation of infection status after weaning were PCR positive further highlighting the imperative need to offer effective interventions to prevent postnatal transmission.

The limitations of the program and this analysis included inability to determine which HIV infected children initiated ART because the HIV care and treatment clinic and EID/PMTCT clinics were run as separate programs. Furthermore, the pilot sites were purposively selected so these results may have limited generalisability. In addition, it was not possible to formally investigate the reasons for the high proportion of loss to follow-up among HIV-exposed infants in this program setting. However, the observations in the first 6 months of implementation, particularly the critical importance of establishing EID services within health systems capable of providing comprehensive continuous care and follow-up of HIV-exposed infant, serve as a critical reference point for informing the scale up of the national EID program in Tanzania.

In conclusion, successful scale-up of EID services in Tanzania and similar settings requires attention to the development of adequate laboratory capacity as well as establishment of systems to ensure appropriate long term follow-up of HIV-exposed and infected infants.

## Competing interests

The authors declare that they have no competing interests.

## Authors' contributions

HN-B conceived the idea for the paper, designed the data capturing systems and tools used for the program, and supervised the data collection and analysis; BW-S, AA, AC, JGG, SM were involved in the implementation of the program, training, and contributed to drafts of the paper; VN conducted the analyses and updates to the reference lists; IM and LFG provided laboratory technical support and contributed to the lab sections in the paper; DN, JJ and EA provided technical oversight for the program implementation and critical review of the paper. All authors have read and approved the final manuscript.

## Pre-publication history

The pre-publication history for this paper can be accessed here:

http://www.biomedcentral.com/1471-2431/10/44/prepub
